# Prevalence of porcine cysticercosis and associated risk factors in Homa Bay District, Kenya

**DOI:** 10.1186/1746-6148-8-234

**Published:** 2012-12-05

**Authors:** Eric E Eshitera, Samuel M Githigia, Philip Kitala, Lian F Thomas, Eric M Fèvre, Leslie JS Harrison, Evalyn W Mwihia, Richard O Otieno, Fred Ojiambo, Ndichu Maingi

**Affiliations:** 1 Department of Public Health Pharmacology and Toxicology, Faculty of Veterinary Medicine, University of Nairobi, P. O. Box 29053, 00625, Nairobi, Kenya; 2 Department of Veterinary Pathology, Microbiology and Parasitology, Faculty of Veterinary Medicine, University of Nairobi, P. O. Box 29053, 00625, Nairobi, Kenya; 3 Centre for Infection, Immunity and Evolution, Institute for Immunology and Infection Research, School of Biological Sciences, University of Edinburgh, Ashworth Laboratories, West Mains Rd, Edinburgh, EH9 3JT, UK; 4 International Livestock Research Institute, PO Box 30709, 00100, Nairobi, Kenya; 5 Royal (Dick) School of Veterinary Studies, The University of Edinburgh, Easter Bush, Midlothian, EH25 9RG, UK

**Keywords:** *Taenia solium*, Cysticercosis, Porcine, Prevalence, Kenya

## Abstract

**Background:**

*Taenia solium* is an important zoonosis in many developing countries. Cysticercosis poses a serious public health risk and leads to economic losses to the pig production industry. Due to scarcity of data on the epidemiology of porcine cysticercosis in Kenya, the present study was conducted to determine the prevalence and risk factors for porcine cysticercosis within Homa Bay district. A cross-sectional survey was carried out in 2010, and a total of 392 pigs were recruited in a household survey, with all being tested by ante-mortem lingual palpation (together with questionnaire data on pig production, occurrence and transmission of porcine cysticercosis, risk factors and awareness of porcine cysticercosis collected from the households from which pigs were sampled). Sufficient serum was collected from 232 of the pigs to be tested for the presence of circulating parasite antigen using a monoclonal antibody-based sandwich enzyme-linked immunosorbent assay (Ag-ELISA).

**Results:**

Seventy six pigs were found positive by the Ag-ELISA (32.8%, 95% C.I. 26.8-39.2%), while by tongue inspection cysticerci were detected in 22/ 392 pigs (5.6% 95% C.I. 3.6-8.4%).

The most important risk factor for porcine cysticercosis in the Homa Bay area was for pigs to belong to a farm where latrine use by members of the household was not evident (OR = 1.9, 95% CI = 1.13–2.37).

**Conclusion:**

The present findings indicate that porcine cysticercosis is endemic in Homa Bay District, and that latrine provision, in conjunction with free-range pig keeping contributes significantly to porcine cysticercosis transmission.

## Background

*Taenia solium* taeniosis-cysticercosis remains a major public health concern in many developing countries of Latin America, Africa and Asia [1]. The occurrence and prevalence of the infection is associated with the practices of eating of raw or undercooked pork as well as poor socio-economic and sanitary conditions. Studies have demonstrated that in endemic areas, *T. solium* porcine infections have been associated with poverty, absence of latrines and free access by scavenging pigs to human faeces [2-4].

Most pigs in central Kenya are raised under a commercial intensive system, but in Nyanza and Western Kenya the extensive management system is predominant, with pigs left free to scavenge. This free-range system is also emerging in certain urban and slum areas of the country. Keeping pigs under a free-range system was actually banned in the 1970s by the Kenya Government and this led to a dramatic decline in the prevalence of porcine cysticercosis within the commercial units that implemented the new regulations [5]. However, the production system is still prevalent in many resource-poor areas, where it is often coupled with poor sanitation, providing perfect conditions for perpetuation of the *T. solium* lifecycle.

In Kenya, little epidemiological work has been done on *T. solium* taeniosis-cysticercosis, and it is assumed to be rare [5,6] and few cases have been reported in pigs during routine meat inspection. The parasite is causing human morbidity however, with 2 cases of human neurocysticercosis having been reported in the recent past [7].

The lingual examination method has been used to detect palpable cysts, which may indicate porcine cysticercosis. However, this method has a low sensitivity, only capable of detecting cysts in heavily infected pigs, reducing its utility as a diagnostic tool [8]. Nonetheless, it has been found to be more readily available and less costly than Ag-ELISA testing. Githigia et al. [5] examined a total of three hundred pigs in South Nyanza and one hundred and seven pigs in Busia, Kenya, using the lingual palpation method and reported prevalence estimates of 10% and 14%, respectively. Acknowledging the low sensitivity of this method it can be expected that these prevalence figures underestimate the true picture of porcine cysticercosis in the regions studied.

There have been no studies to establish the prevalence of porcine cysticercosis and its risk factors in Homa Bay District. This study investigated the prevalence of porcine cysticercosis and the potential risk factors of *Taenia solium* taeniosis and cysticercosis in Homa Bay District using both lingual palpation and the more sensitive Ag-ELISA method.

## Methods

### Study area

The study was conducted in Homa Bay district in Nyanza province of Kenya. The study site was chosen due to an observed predominance of free range pig husbandry and because no previous studies on porcine cysticercosis had been conducted in the district. Homa Bay district is located approximately 500 km from Nairobi within the Lake Victoria Basin. The district covers an area of 1160 Km^2^ and is made up of seven administrative divisions namely: Asego, Riana, Rangwe, Nyarongi, Kobama and Ndhiwa. The altitude ranges between 1163 m and 1219 m above sea level, most parts of the district receive 500–1000 mm mean annual rainfall, in a bimodal pattern falling between April to May and November to December and temperatures range between 17.1°C and 34.8°C. The total human population of Homa Bay District was estimated at 963,794 people with the majority of residents being subsistence smallholder farmers [9].

### Sample size determination

The sampling strategy was random multistage whereby all seven divisions were selected to increase the geographic spread. Then two locations per division were randomly selected thus resulting in fourteen locations. One third of the sixty six sub-locations within the selected fourteen locations were randomly selected at the second stage resulting in 22 sub-locations. A further one third of the 126 villages within the selected sub locations were subsequently randomly selected at the third stage resulting in 42 villages, with the number of villages per division being proportionate to the pig population in the division. All pig-keeping households in the selected villages were to be included in the study. The households sampled were proportionately distributed in the divisions of the district with the district with a higher pig population having more households being sampled. The pig-keeping households were established with the help of the veterinary extension officers and the local administration. The sample size of households and pigs that were to participate in the study was computed using the formula n = Z_α_^2^pq/L^2^[10]. Where, n is the required sample size, Z_α_ = 1.96 is the standard normal deviate at 5% level of significance, p is the estimated prevalence, q = 1-p, and L is the precision of the estimate. Setting p = 0.1 [5] and L at 5%, the required sample size was 138 households. In households with more than two pigs, a proportion of the pigs were randomly selected for sampling. For instance where there were three pigs two were sampled and where there were five, three were sampled.

### Sample collection

Sampling of animals was carried out with authority from the Republic of Kenya Department of Veterinary Services (Ministry of Livestock Development, Nairobi, Kenya). Lingual examination and blood sampling on pigs were conducted humanely and oral consent was sought from the owner before commencement of the study on a pig.

All pigs that met our selection criteria were examined for the presence of *T. solium* cysticerci by lingual palpation. Briefly, the pig was firmly restrained and a pig snare was used to stabilize the head and a hard wooden stick used to open the mouth. Using a piece of cotton gauze for grip, the tongue was pulled out, examined and palpated all along its ventral surface for the presence of cysticerci. Then, about 5 ml of blood were obtained from the anterior vena cava using vacutainers with clot activators. The blood was transported on ice to the laboratory and allowed to clot at 4°C. To obtain serum, the clotted blood was separated by decanting, and serum was dispensed into 2 ml labeled aliquots and stored at −20°C until use.

### Household questionnaire

A structured questionnaire with both closed and open-ended questions was administered to a member of the selected household who was familiar with the day to day raising of the pigs owned by that household. The household questionnaire was pretested by administration to individuals from 12 pig keeping households selected from two divisions in Homa Bay District. The pretested households were then excluded from the actual study.

The questionnaire survey obtained data on pig production and husbandry and risk factors for occurrence of porcine cysticercosis and *T. solium* cysticercosis and taeniosis. The questionnaire was written in English and administered by the principal investigator, through personal interviews with the respondents. Where the person being interviewed could not understand English or Swahili, the local *dholuo* language was used with the assistance of an interpreter. Risk factors considered in this study included housing of pigs, lack of evidence of latrine use (this was determined through visual appraisal by the investigator through checking for aspects such as a beaten path to the latrine, presence of a lockable door, evidence of cleaning), history of shedding tapeworm segments in stool by a household member, history of epilepsy in a household member, pork inspection; these are similar to those reported by [11-14].

### Enzyme-linked immunosorbent assay for detection of circulating antigens

This procedure was carried out at the International Livestock Research Institute (ILRI), Nairobi. The HP10 antigen was detected by Ag-ELISA as described previously by Harrison *et al.*[15]. The procedure was as follows: 100 μl of a 10 μg/ml solution of 50% saturated (NH_4_) _2_S0_4_ precipitate McAb HP10 diluted in carbonate-bicarbonate buffer 9.6 pH (Sigma C3041) was added to each of the wells of a flat bottomed Immunlon® 4HBX ELISA plate and the plate was incubated overnight at 4°C. The wells were washed out twice with washing solution (0.9% NaCl with 0.05% Tween®20 (Sigma P1379)), 200 μl of phosphate buffered saline (PBS) 7.3 pH (Sigma P4417) / 1% Bovine Serum Albumin (BSA) (Sigma A4503) / 0.05% Tween®20 was added to each of the wells and incubated at room temperature for 1 hour to block any non reacted sites on the plate. The plates were then washed three times with washing solution. 100 μl of undiluted sera was then added to each well, with each sample running in duplicate. The plate was incubated for 1 hour at 37°C, followed by emptying and washing three times. Biotinylated-McAb diluted 1:2,500 in PBS/BSA/Tween®20 was added at 100 μl/well, covered and incubated for 1 hour at 37°C followed again by washing three times. Streptavidin Peroxiase (sigma S5512) conjugate diluted 1:10,000 (i.e. 0.1 μg/ml) in PBS/BSA/Tween®20 added at 100 μl per well, covered and incubated for 1 hour at 37°C. After a further three washes 100 μl 3,3’, 5,5’- Tetramethylbenzidine substrate (Sigma T8665) substrate was then added to each well and incubated at room temperature for 15 minutes. The reaction was then stopped with 100 μl of 0.2 M H_2_SO_4_ per well and read at 450 nm on an ELISA plate reader. A sample was considered positive if the mean OD value of the duplicates was higher than the cut-off value, which was calculated based on the mean of the OD plus 3 Standard Deviations of the 5 samples from non-exposed controls. In this case, controls were piglets from a clean, indoor pig unit in Kitengela in Kajiado District, Kenya.

### Statistical analysis

The questionnaire data were combined with pig serology data in a MS Excel (Microsoft Corporation) spreadsheet and then exported to SPSS (PASW Statistics 18) software for analysis. Summary statistics were generated using the same software. For the purpose of modeling these data, explanatory variables were first explored for any associations with the serology result using χ2 test. A liberal *p*-value (0.15) was used to determine significance [16]. The strength of the associations was determined using the odds ratio (OR). Correlations between the explanatory variables were assessed to identify highly correlated variables (>0.5) this was then followed by a backwards elimination logistic regression proceeding from the variables with the highest *p*-values to arrive at the most parsimonious model. A threshold *p*-value of 0.1 was used in order to include only those variables that are strongly significant.

The factors that were considered in the analysis as risks associated with porcine cysticercosis at household level were: history of tapeworm shedding by a household member, absence of evidence of latrine use, pork inspection, history of epilepsy in a household member and presence of free range pigs. The full model used for the multivariate analysis was:

(1) $$\begin{array}{rl} Yi=\beta_{0} & +\;\beta_{1}\left(\text{lack}\;\text{of}\;\text{pig}\;\text{housing}\right)+\beta_{2}\left(\text{pork}\;\text{inspection}\right)\\ & +\beta_{3}\left(\text{lack}\;\text{of}\;\text{evidence}\;\text{of}\;\text{latrine}\;\text{use}\right)\\ & +\beta_{4}\left(\text{shedding}\;\text{tapeworm}\;\text{segments}\right)\\ & +\beta_{5}\left(\text{epilepsy}\;\text{in}\;\text{household}\right)+\varepsilon_{i} \end{array}$$

Of the variables introduced in the logistic regression model lack of evidence of latrine use remained the only significant variable associated with seropositivity for porcine cysticercosis in the study (Table 1). The final model was: Y*i* = β_o_ + β_3_ (lack of latrine use).

**Table 1:** Bivariate analysis of factors associated with a positive Ag-ELISA test result in Homa Bay District, Kenya, 2010

**Risk factor**	**Level**	**Ag-ELISA**		**Prevalence (%)**	**Risk ratio (RR)**	***p *- value**
		**+**	**−**			
**Absence of evidence of latrine use**	Yes	29	50	36.7	1.6	< 0.05
	No	36	118	23.4		
**Pork inspection**	Yes	21	40	34.4	1.3	> 0.05
	No	44	128	25.6		
**History of tapeworm shedding**	Yes	61	148	29.2	1.8	> 0.05
	No	4	20	16.7		
**History of epilepsy**	Yes	18	42	30	0.9	> 0.05
	No	50	116	30.1		

## Results

A total of 392 pigs were examined from 299 households proportionately distributed in the divisions of the District. Most of the sampled pigs were of no determinable breed, over half of the pigs sampled were female (59%) and approximately 75% were less than 12 months old.

The majority of farmers left their pigs to scavenge during the day and during the dry season, with only 1.6% of farmers practicing total confinement of their pigs. The pigs were mainly fed on kitchen left-overs, sweet potato tubers and vines, guavas, cassava, brewers mash and pasture. A small proportion of farmers (4%) had slaughtered pigs at home, but even of those who slaughtered pigs away from the home, the majority (67%) had their pigs slaughtered without inspection.

Women take a leading role in pig keeping, with 64% of respondents to this survey being female. Pork eating was common, with 68% of respondents eating pork at least once a year, however no respondent recalled having ever seen a cyst in a pig and the majority (99.7%) of respondents did not know how pigs acquired this infection.

All 392 pigs recruited to this study underwent an ante mortem lingual palpation and 232 pigs had sufficient sera to test with the HP10 antigen capture ELISA. Results of the lingual palpation and HP10 Ag-ELISA can be found in Table 2.

**Table 2:** **Prevalence estimates for *T. solium *based upon lingual palpation and antigen ELISA**

**% Pigs with positive lingual palpation**	**% Pigs testing positive with HP10 Ag-ELISA**	**% Households with at least 1 pig testing positive on HP10**
22/392 5.6%	76/232 32.8%	140/299 46.9%
(95% CI 3.6-8.4%)	(95% C.I. 26.8-39.2%)	(95% CI. 39.6%–54.2%)

Over half of the households (52%) reported having no latrine and in the univariate analysis, this was the only risk factor considered which was significantly (*p* < 0.05) associated with circulating antigens of *T. solium* (χ2 **=** 4.59, *p* = 0.032). The prevalence of circulating antigens in pigs from households with no evidence of latrine use (36.7%), was significantly (p < 0.05) more than the prevalence in households with evidence of latrine use (23.4%). Pigs from households without evidence of latrine use were 1.6 times more likely to test positive for circulating *T.solium* antigens relative to pigs from households with evidence of latrine use as shown in Table 1.

The population attributable risk (PAF) for absence of evidence of latrine use was 54.54% indicating that 54.54% of the Ag-ELISA positive results in the pig population of Homa Bay District was due to lack of latrine use.

Of the variables introduced in the logistic regression model lack of evidence of latrine use remained the only significant variable associated with seropositivity for porcine cysticercosis in the study with an odds ratio (OR) of 1.9 (95% CI; 1.05,3.43) as shown in Table 3.

**Table 3:** Description and contingency test results for explanatory variables used in logistic regression analysis

**Category**	**Variable**	***β *coefficients**	**Chi-square**	***p*-value**
**Household characteristics**	**Absence of evidence of latrine use**	0.626	4.59	<0.05
	**Pork inspection**	0.419	1.74	>0.05
	**Tapeworm shedding**	0.479	1.67	>0.05
	**History of epilepsy**	- 0.684	0.89	>0.05

## Discussion

This study has investigated the prevalence and the potential risk factors associated to *T. solium* cysticercosis in pigs in Homa Bay district. The overall mean prevalence based on lingual palpation was 5.6% while by detection of circulating antigens the mean was 32.8% indicating that porcine cysticercosis is highly prevalent in this area. This figure is within a similar range to those reported elsewhere in endemic areas of Africa using Ag-ELISA. Prevalence estimates include, 23.3% in the Eastern, Southern and Western provinces of Zambia [17], 34.9% in Angonia district, Mozambique [18], 38.4% in Congo [19].

The high prevalence of cysticercosis found in this study is likely to relate to the popularity of free-range pig keeping, as the vast majority (98%) of pigs where kept under such a system for all or part of the year, despite its illegal status. This coupled with poor levels of latrine use sets up a perfect situation for perpetuation of the parasitic lifecycle, with pigs being exposed to infective eggs and proglottids in human faecal material.

Half (52%) of the households in this study had no latrines. Previous studies in Tanzania have reported an association between not having a farm latrine and occurrence of porcine cysticercosis [20]. In this study, absence of a toilet or latrine supported by evidence of no use in homesteads with latrines was similarly the only significant risk factor for porcine infection identified.

The lower prevalence estimate by lingual palpation compared to Ag-ELISA is due to the lower sensitivity of the lingual palpation method. The HP10 Ag-ELISA has been reported to have an estimated sensitivity in pigs of 70.4% (52.7-84.7) [21], in comparison to an estimated sensitivity of between 7% (95% CI 0.8-15%) [21] and 16% (95% C.I. 5-34%) [22] for lingual palpation.

Several factors indicate that the community of Homa Bay district are at high risk of acquiring a *T.solium* infection. Approximately three-quarters of respondents reported eating pork at least once a year, Over 60% of pork slaughtered in the area was not inspected for cysticercosis, which combined with the high prevalence of the parasite in this population indicates that many of the pork meals consumed will be infected with *T.solium*. Infective eggs from *T.solium* can be passed to humans through faecal-oral contamination, be that by hands, food products or water. Less than 50% of respondents in this study reported boiling their drinking water, increasing the risk of acquiring many waterborne illnesses as well as *Taenia solium.*

It is interesting to note that over half of the respondents in this study were female. This was also seen in Tanzania [18,23] and indicates that pig production is influenced strongly by women. It is important to remember this when designing control interventions, to ensure that female famers are targeted as strongly as their male counterparts.

Homa Bay district is a deprived area of Kenya, with low levels of employment, limited access to clean water, toilet facilities and refuse disposal. There is a growing demand for pork products and pig production can be utilized to improve the economic and nutritive well-being of farmers in this area. However, a free-range system, which exposes pigs to many diseases, including the zoonotic *T. solium* can result in poor profitability of these ventures. A lack of accessible veterinary services in the area is also a strong constraint upon the industry, as was reported by Mutua *et al.*[24] and Kagira *et al.*[14].

## Conclusion

*T.solium* is a parasite of serious public health importance in the Homa Bay area; improving latrine provision in this area would go a long way towards controlling this parasite and to encourage the development of a viable, prosperous, pig production industry.

## Competing interest

The authors declare that they have no competing interest.

## Authors’ contributions

EE designed and carried out the study, NM and SG provided guidance on parasitology aspects of the study and data collection, PK provided guidance on epidemiology and statistical aspects of the study, LFD was involved in analyzing the samples in the laboratory, assisted in data interpretation and in drafting the manuscript, EMF was involved in data analysis and drafting the manuscript, LJSH provided technical assistance and oversight for the laboratory analysis. EM assisted with statistical analysis and proof reading, FO translated the questionnaire into the local Dholuo language RO assisted the team in the field with sample collection and preparation. All authors read and approved the final manuscript.
